# Arthroereisis: Treatment of Pes Planus

**DOI:** 10.7759/cureus.21003

**Published:** 2022-01-07

**Authors:** Abdullah Ghali, Aum Mhapankar, David Momtaz, Brandon Driggs, Ahmed M Thabet, Amr Abdelgawad

**Affiliations:** 1 Surgery, University of Texas Health Science Center at San Antonio, San Antonio, USA; 2 Orthopaedics, University of Texas Health Science Center at San Antonio, San Antonio, USA; 3 Orthopaedics, Texas Tech University Health Sciences Center, El Paso, USA; 4 Orthopaedics, Maimonides Medical Center, Brooklyn, USA

**Keywords:** flatfoot, deformity, pes planus, arthroereisis, ankle and foot

## Abstract

Arthroereisis is a surgical procedure primarily used to treat flexible pes planus (flatfoot) in pediatric and young adult patients. The principal goal of subtalar arthroereisis is to relieve pain and restore function. This is primarily done by restoring the medial foot arch without fusing the subtalar joint and without requiring a long recovery period needed after osteotomies. Although the procedure can be performed in isolation to treat flexible flatfoot, it can also be performed as an ancillary in the treatment of tarsal coalition, posterior tibial tendon dysfunction, and accessory navicular syndrome. Various implants and multiple surgical techniques exist for arthroereisis, such as the sinus tarsi implant and calcaneo-stop. The type of device and the surgical approach to proceed with are based on the surgeon’s discretion rather than an evidence-based protocol. Multiple complications can arise from subtalar arthroereisis, most commonly sinus tarsi pain. Currently, there is a dearth of quality clinical data and evidence on the long-term outcomes and complications of arthroereisis. This lack of literature for a commonly performed procedure validates the need for future studies to better guide a standard protocol, reach consensus on well-defined indications and contraindications, provide expected complications, and improve the practice of evidence-based medicine.

## Introduction and background

Anatomy of the subtalar joint

**Figure 1:**
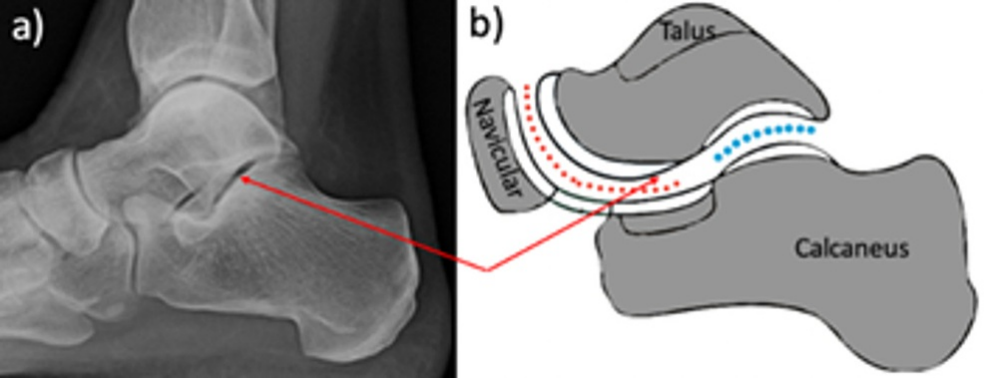
The subtalar joint. Articulation of the calcaneus with the talus bone can be seen. The red arrows show the subtalar joint on a lateral X-ray and illustration. The red dashed lines represent the talocalcaneonavicular joint while the blue dots represent the talocalcaneal joint.

The subtalar joint articulates the talus, calcaneus, and navicular bones of the foot (Figure 1). The functional subtalar joint is anatomically separated into the talocalcaneal joint and talonavicular joint, with the sinus tarsi and tarsal tunnel surrounding the subtalar joint laterally and medially [1]. The superior and inferior surfaces of the sinus tarsi are formed by the talar neck and calcaneus, respectively. The talocalcaneonavicular joint articulates in a ball-and-socket manner. The ligaments of the talocalcaneonavicular joint include the spring, dorsal talonavicular, and part of the bifurcate ligament [1,2].

The subtalar joint holds three articular facets on the superior calcaneus and inferior talus, all of which vary greatly in size and shape. Functionally, during normal gait, supination of the subtalar joint locks and transforms the foot from a position for shock absorption to a mechanical lever that aids the foot in propelling the body forward. Proper alignment of the articular facets, ligaments, talus, navicular, and calcaneus on the subtalar joint axis is essential to the physiology of all steps of human gait [1].

Pathology of flatfoot

**Figure 2:**
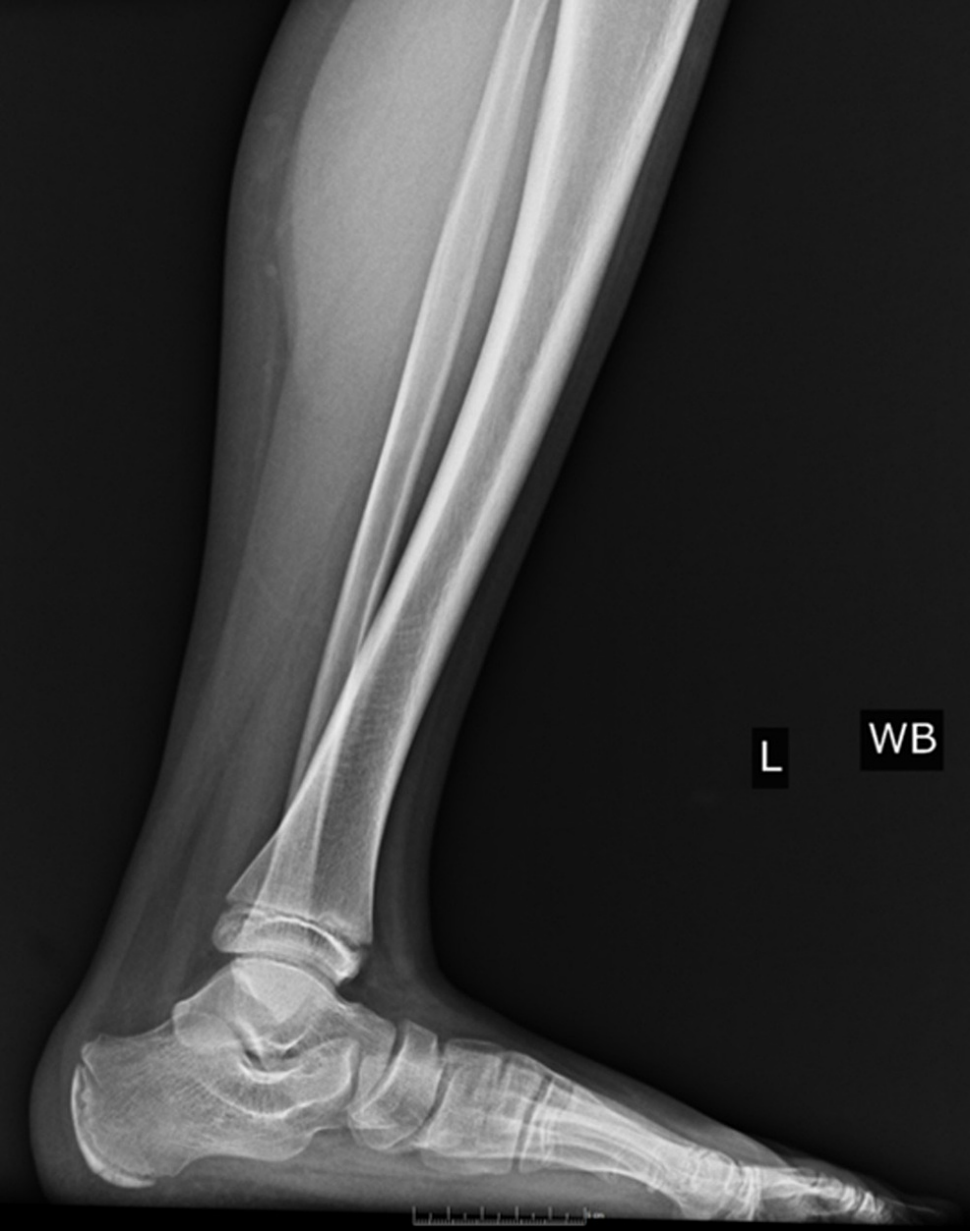
Lateral preoperative left foot weight-bearing X-ray.

**Figure 3:**
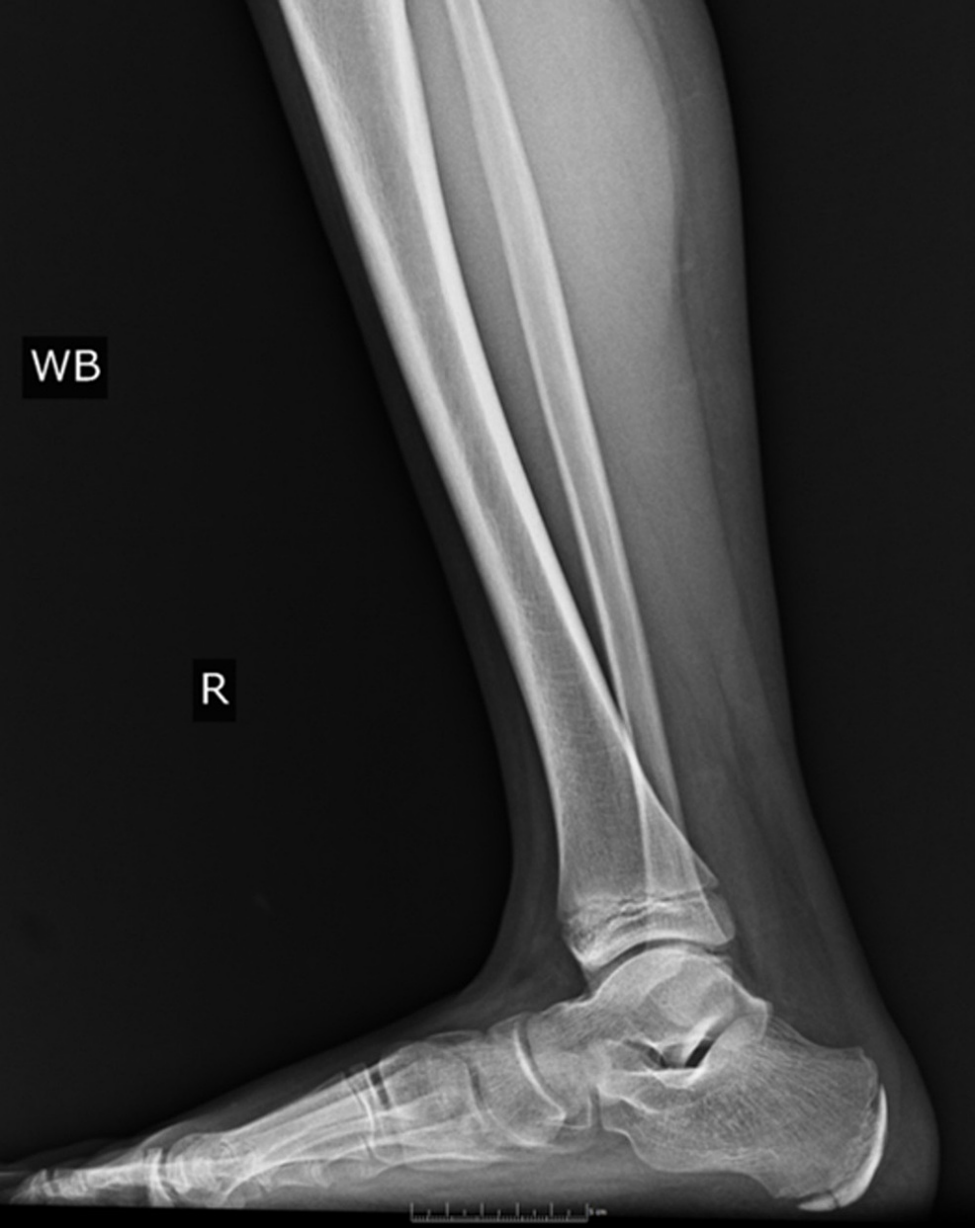
Lateral preoperative right foot weight-bearing X-ray.

**Figure 4:**
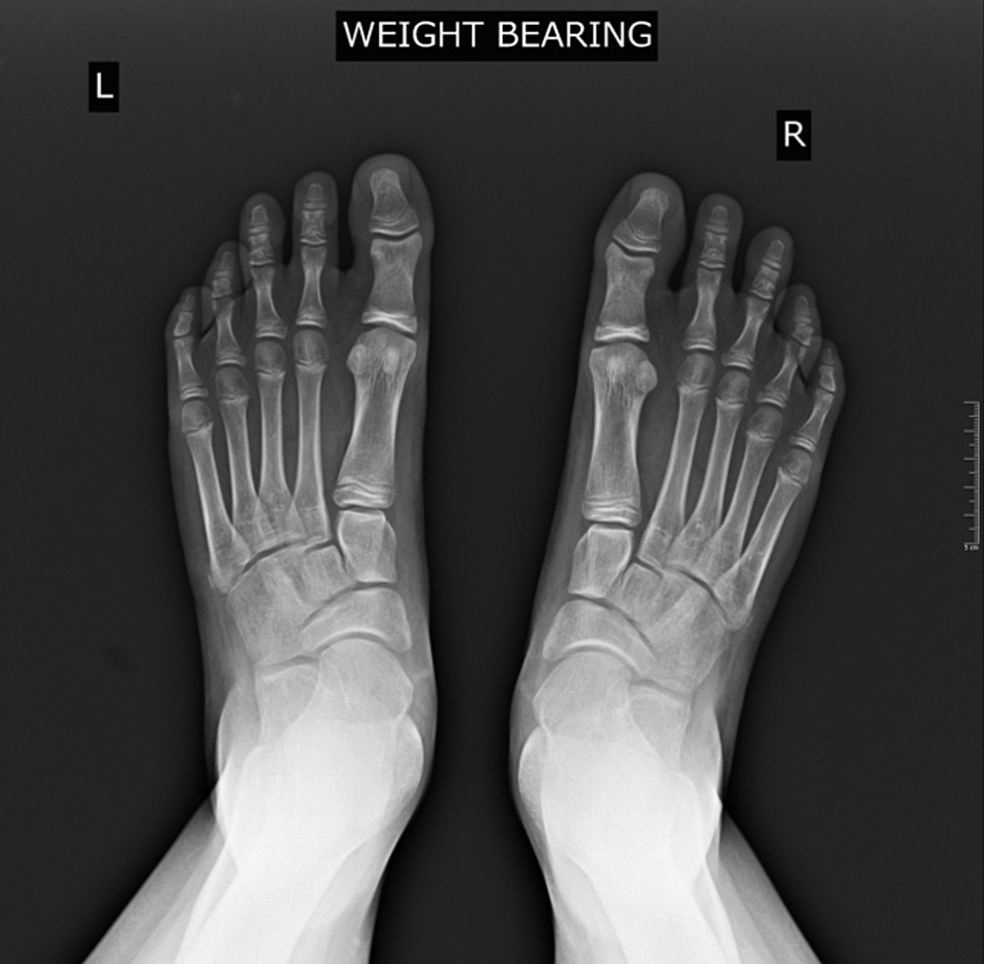
Anteroposterior preoperative bilateral weight-bearing feet X-ray.

Pes planus, the primary condition for which arthroereisis is performed, occurs due to the abduction of the forefoot, excess subtalar eversion, and resultant loss of the medial longitudinal arch of the foot [3]. Pediatric flatfoot may be categorized into rigid and flexible flatfoot. Flexible flatfoot results from flattening of the foot’s medial longitudinal arch. A key feature is the restoration of the arch with tiptoeing or dorsiflexion of the big toe. In many cases, flexible flatfoot can be physiologic and makes up approximately 95% of pediatric flatfoot cases [4]. Conversely, a rigid flatfoot is non-physiologic and constitutes a flattened arch in both non-weight-bearing and weight-bearing stances [5]. Figures 2-4 display weight-bearing X-rays of a patient with pes planus preoperatively.

Treatment

**Figure 5:**
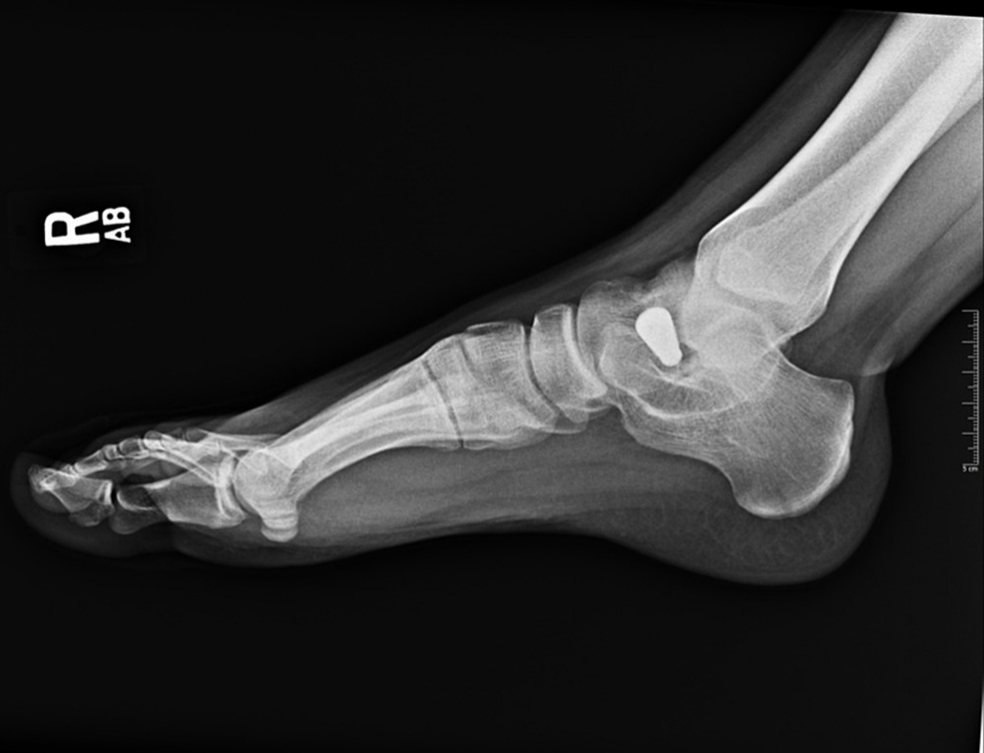
Lateral postoperative right foot weight-bearing X-ray.

**Figure 6:**
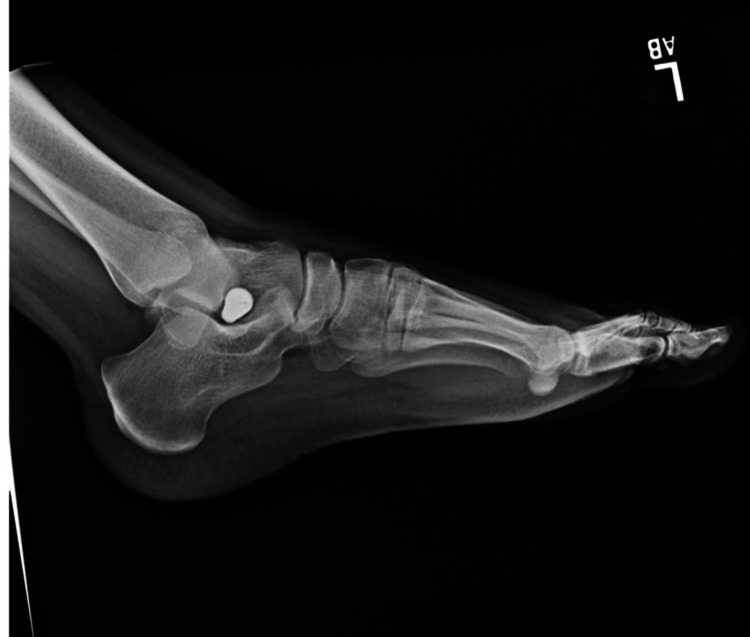
Lateral postoperative left foot weight-bearing X-ray.

**Figure 7:**
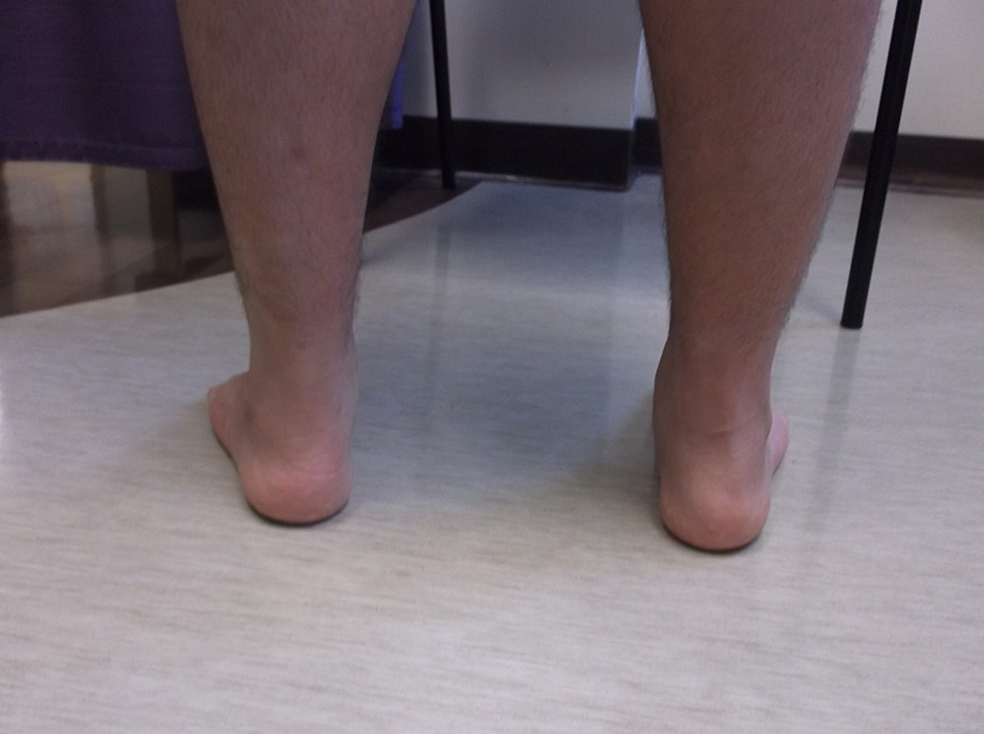
Clinical image of the postoperative bilateral feet.

**Figure 8:**
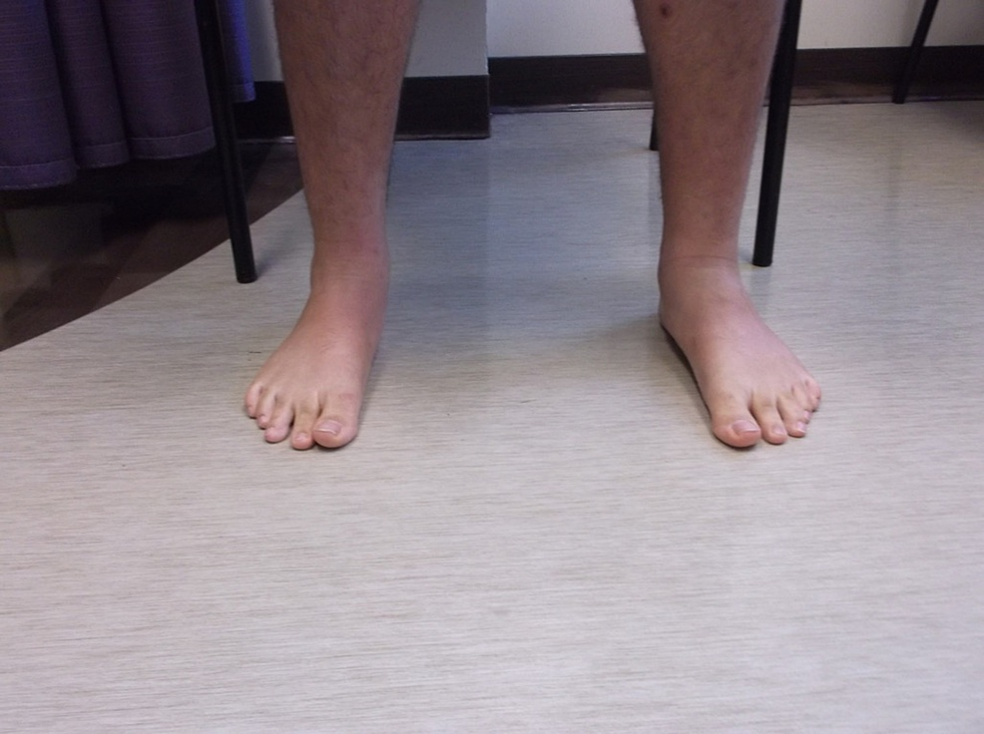
Clinical image of the postoperative bilateral feet.

The initial management of pes planus includes exploring all conservative measures as many pediatric patients go on to have no long-term issues with minimal intervention. Conservative measures include rest, anti-inflammatory medications, physiotherapy, and orthotics. However, if pain persists and there are functional ambulatory difficulties affecting daily life, operative treatment may be indicated [3]. Numerous surgical options are commonly adopted. The first option includes soft tissue modifications, such as Z lengthening of the Achilles tendon or flexor digitorum longus, flexor hallucis longus, and peroneus brevis. This may be done independently or in conjunction with another surgical method because these muscles can exacerbate flatfoot if they are spastic or shortened [6]. Another option includes medializing, osteotomy aiming at the lateral calcaneal column, or medial cuneiform opening wedge osteotomy. The principle behind osteotomy involves correcting the valgus deformity of the hindfoot by altering the positioning of the calcaneal bone itself [6]. Additionally, arthrodesis is a salvage technique that is seldom indicated in the pediatric population. Arthrodesis involves permanent fusion of the talocalcaneal, talonavicular, and calcaneocuboid joints. It achieves a low rate of recurrence with the drawback of loss of mobility and altered biomechanics of the foot. The final surgical option is a type of arthroereisis procedure referred to as the calcaneo-stop screw, which is the focus of this review. This involves inserting a screw that acts as an obstacle to prevent hypermobilization of the subtalar joint. This stabilization of the subtalar joint allows remodeling of the joint surface and tightening of the capsules and tendons (such as extensor digitorum longus, extensor hallucis longus, and peroneus longus/brevis), a change which is only possible in pre-pubescent children [6]. Figures 5, 6 display postoperative arthroereisis weight-bearing X-rays of a patient with pes planus. Figures 7, 8 display the clinical pictures of a patient postoperatively showing improvement in his medial arch.

History

The initial discourse on surgical alteration of the tarsi and the subtalar joint arose in 1946 when Chambers suggested correcting flatfoot by placing a wedge upon the superior surface of the calcaneus to restrict the anterior displacement of the talus from the calcaneus [7]. The first surgeon to insert a cortical bone wedge graft into the sinus tarsi was Stefán Haraldsson in 1962. In doing so, he limited subtalar eversion to treat his patients with pes planus. LeLièvre from France was the first to coin the term “arthroereisis” in 1970, at the time detailing a bone graft insertion into the sinus tarsi that is fixed with a staple [8]. Soon after, in 1974, Subotnick advanced the idea of placing a synthetic silicone implant into the sinus tarsi to better support the articulation of the talus with the calcaneus into the proper position [9]. Thus, the concept of the modern-day arthroereisis procedure was beginning to materialize. In 1992, the recent model of arthroereisis began to take its place when Antonio Viladot outlined a cup-shaped silicone implant that could be implanted into the sinus of tarsi. In his study, Viladot performed this procedure on 234 children as a treatment for flatfoot, with a 99% success rate (normalization in 55.5% of feet and improvement in 43.7% of feet). Despite this extremely high success rate, he reported some complications, such as infection and pain [10]. Since arthroereisis first rose onto the podiatric and orthopedic surgery landscape, a myriad of improvements have been suggested to alter the implant’s shape to create a block, screw, or cap, and material to construct it out of polyethylene, titanium, or silastic [8].

## Review

Design classification

The modern classification of subtalar implants used in the arthroereisis procedure is based on four critical aspects of the device: shape, anatomic orientation within the tarsal sinus, location the device is anchored within the sinus, and mechanism of talar stabilization [11]. Implants currently on the market can be classified into two major types, namely, type I and type II, with type I further subclassified into type IA and type IB. Type IA is cylindrical with a lateral-to-medial orientation within the tarsal sinus, is anchored by soft tissue, and functions by impediment within the sinus. Type IB differs from IA only in its shape; it has a conical shape, with other features being identical. Type II is unique in that its geometry is modeled after the anatomy of both the sinus and the canalis portions of the tarsal sinus, with the lateral portion conical and the medial portion cylindrical in shape [11]. The anterior-most end of type I devices, when inserted fully, reaches the “cruciate pivot point,” which is along the longitudinal talar bisection line. This pivot point is precisely the point at which excess anterior-medial-plantar talar displacement should be minimized [11]. Because type I devices reach only up to this point, they may not always provide the desired talar stability. Type II devices, however, not only have a conical portion on the lateral portion of the device but also a cylindrical portion extending further medial to that, into the canalis portion of the tarsal sinus. The tapered, conical portion of the implant stabilizes the device in the sinus, while the cylindrical half of the device extends slightly beyond the longitudinal talar bisection line [11]. In this way, type II devices offer the closest anatomical fit, which has been shown to provide a more uniform distribution of force and optimized biomechanics [12].

The popularity of the procedure

The subtalar arthroereisis was widely adopted during the 1970s but has since fallen in and out of practice [3]. Despite losing some degree of pervasiveness, a large proportion of providers perform arthroereisis according to a 2013 survey of the American Orthopedic Foot & Ankle Society (AOFAS). Overall, 69% of AOFAS respondents reported performing subtalar arthroereisis. According to the 2013 study, the primary reason for the discontinuation of arthroereisis was its low success rate, as stated by 68% of respondents who no longer performed arthroereisis [13]. More recently, the procedure has started to gain more attention and more surgeons have started to perform arthroereisis. With patients looking for treatment options on the internet and social media, patients have started to ask their surgeons about the “minimally invasive” solution.

Anatomical and mechanical effects of arthroereisis

Sinus tarsi implants placed during subtalar arthroereisis procedures have not been found to alter the anatomy of the subtalar joint or press against articular joint cartilage. With the exception of the subtalar peg, the implant rests entirely within the sinus tarsi [14]. A systematic review of over 24 articles and 2,550 feet operated on showed postoperative radiological ranges trending toward the normal anatomical values. The anteroposterior (AP) talar first metatarsal, AP talar calcaneal, and lateral talar first metatarsal angles, as well as the calcaneal incidence, normalized after arthroereisis. Kinematic measurements also showed progression toward anatomical normality, with a mean reduction in hindfoot valgus of 8.1 degrees [3]. Pedobarographic analysis comparing postoperative feet after arthroereisis with a control group of normal feet showed normalized contact area between medial and lateral midfoot in the arthroereisis group compared to the control group. In addition, load distribution in the forefoot was improved and normalized in the patients after arthroereisis [15].

Surgical indications

The primary indication for subtalar arthroereisis is the non-fusion treatment of more symptomatic forms of pediatric flexible pes planus (flatfoot). Although there is no consensus on the treatment of pes planus, one of the main surgical recommendations to relieve pain and improve function is subtalar arthroereisis [8]. Subtalar arthroereisis can also be a promising option in adults for correction of flexible flatfoot secondary to posterior tibial tendon dysfunction. Some studies have compared other techniques such as lateral calcaneal lengthening to arthroereisis and concluded that arthroereisis resulted in rapid symptom relief, early weight-bearing capacity, and is a less invasive procedure. However, the literature is relatively limited in the adult population [16,17].

Tahririan et al. differentiated between two subtypes of flexible pes planus in pediatric and young adults to help guide the treatment plan. First, those with marked forefoot abduction can be better managed with lateral column lengthening (either Evan’s-type calcaneal osteotomy or cuboid lengthening). Second, those with limited forefoot abduction but excessive subtalar eversion can be better managed by arthroereisis [17].

Contraindications

Subtalar arthroereisis has mainly been used to correct flexible flatfoot. In fact, rigid pes planus may contraindicate the use of arthroereisis as the primary surgical procedure [3]. Other contraindications include active infection, prior sinus tarsi surgery or trauma, and advanced arthrosis of the subtalar joint [12].

Sinus tarsi implant technique

**Figure 9:**
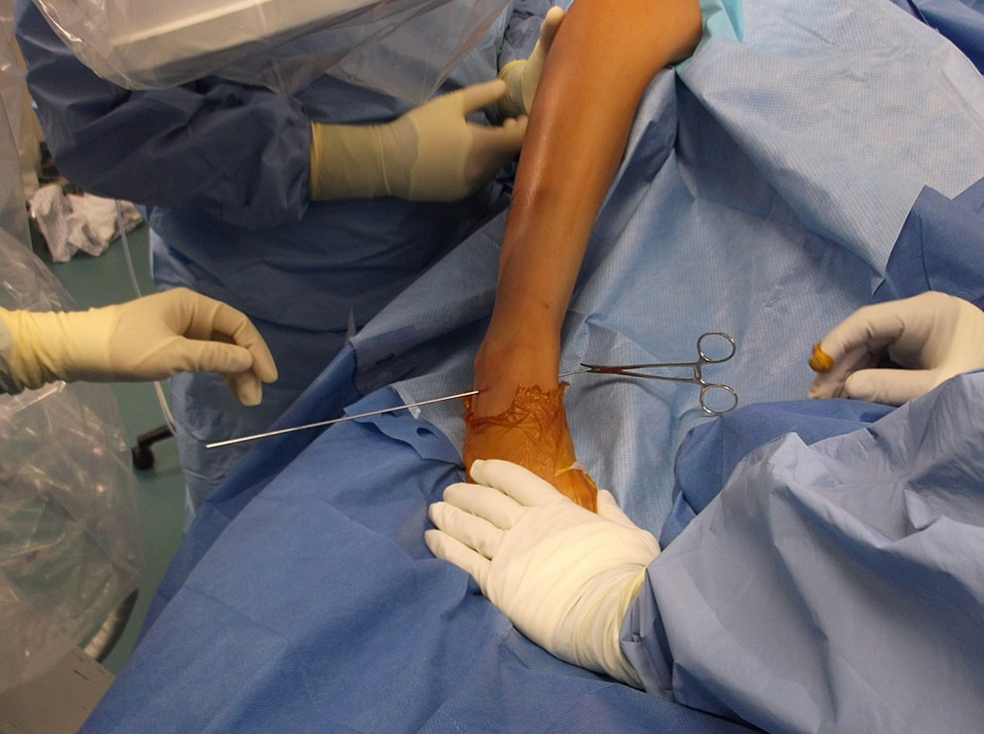
Operative image displaying guidewire placement with a hemostat clipped medially.

**Figure 10:**
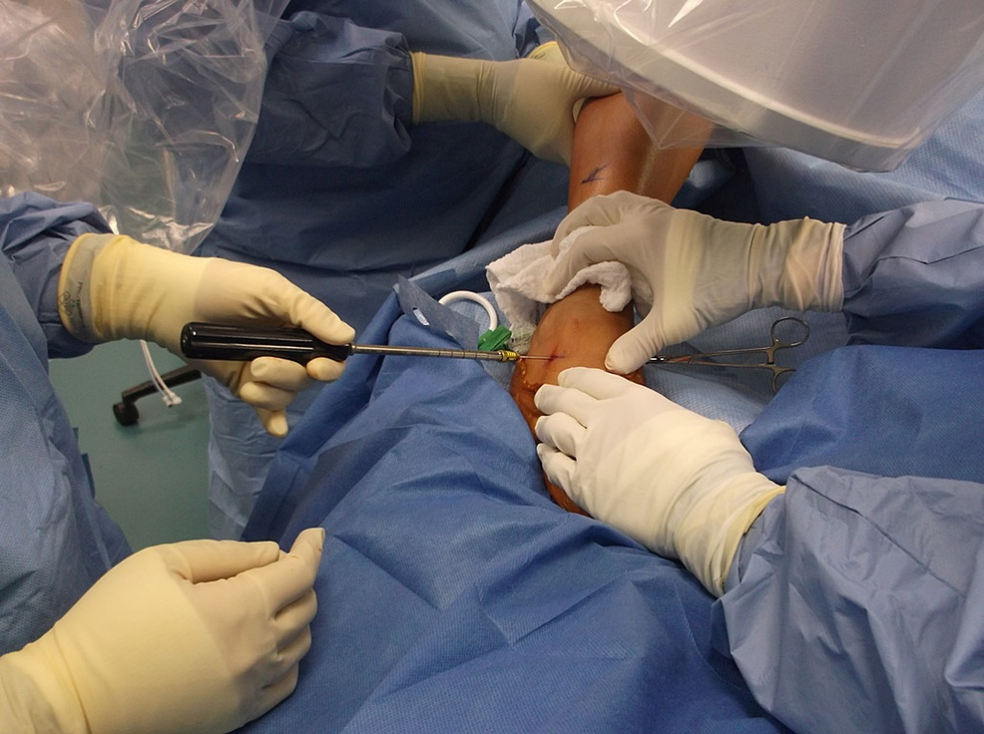
Clinical image of operative arthroereisis.

**Figure 11:**
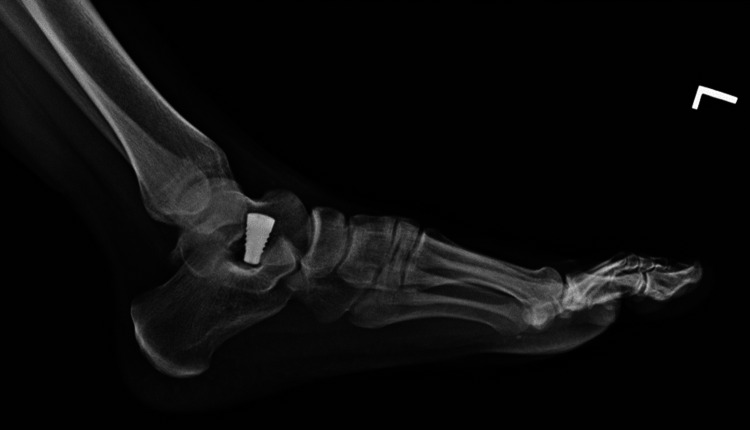
Postoperative lateral left foot X-ray with sinus tarsi implant.

**Figure 12:**
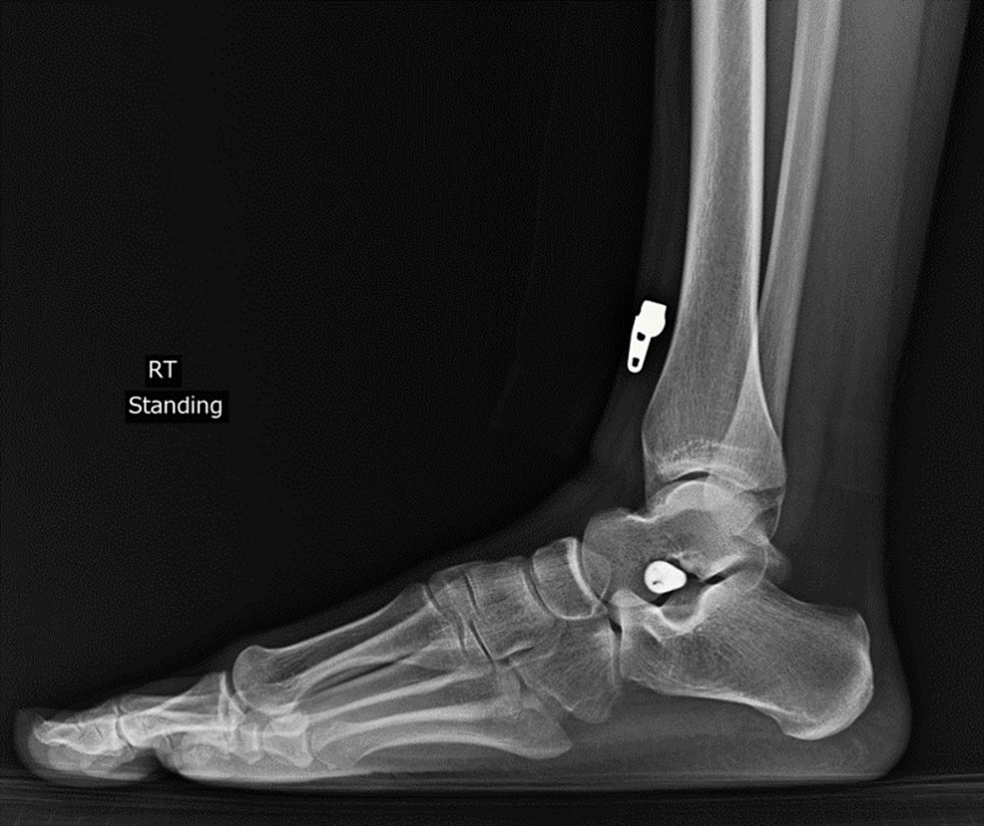
Postoperative lateral right foot X-ray with sinus tarsi implant.

There are multiple techniques and approaches to arthroereisis surgery, which is typically completed within 20 minutes [18]. The procedure can be done under general, regional, or local anesthesia. However, in children, most cases are performed under general anesthesia to ensure that the child stays still. As the procedure is performed percutaneously, a tourniquet is not needed. With the patient in a supine position, the foot undergoing the operation is rotated internally. A 1 cm incision is made over the sinus tarsi along the relaxed skin’s tension lines. A hemostat is used to spread the tissue to the level of the sinus tarsi. Subsequently, percutaneous insertion of the guide laterally to medially occurs across the floor of the sinus tarsi. The direction is from lateral anterior distal to medial posterior proximal (the direction of the sinus tarsi). The guide pin is pushed to the medial side, and a hemostat is used to clamp the guidewire on the medial side to avoid bulling back of the wire during trials, as seen in Figures 9, 10. The guide pin crosses superior to the posterior tibial tendon and inferior to the medial malleolus. Sizing guides are inserted, followed by trial implants. The goal is to limit excess eversion (approximately 5 degrees of eversion from neutral). There is a need to avoid the temptation to “overstuff” the sinus tarsi with a large implant. The implant is placed medially to the lateral edge of the calcaneus by approximately 1 to 1.5 cm, and its positioning can be verified with AP images. Laterally, the implant should be seen planted on the floor of the sinus tarsi [19]. If any deformity is present in the other foot, bilateral arthroereisis can be performed. After surgical correction, dorsiflexion of the foot is performed with the knee extended. If dorsiflexion is limited, subcutaneous lengthening of the Achilles tendon is required until the foot can be dorsiflexed to 10 degrees [20]. Figures 11, 12 demonstrate postoperative weight-bearing X-rays of a patient with bilateral sinus tarsi implants.

Calcaneo-stop technique

One of the methods of arthroereisis is known as calcaneo-stop. First introduced in 1995 by Alvarez, the calcaneo-stop method aims to limit subtalar joint motion via a screw inserted into the calcaneus. This approach to arthroereisis is less expensive and results in similar improvements in pain and function. Similar to the previous technique, calcaneo-stop utilizes the sinus tarsi for entry [18]. In this procedure, a tourniquet is wrapped around the thigh. The aim is to insert the calcaneo-stop 1 cm medial to the lateral border of the calcaneus within the sinus tarsi. Keeping the calcaneo-stop screw as vertical as possible, the screw is inserted with proper care to ensure steady placement into the insertion point. The head of the screw should impose on the lateral aspect of the talus to prevent eversion of the subtalar joint. After the stitches are placed, dorsiflexion is tested, similar to the sinus tarsi procedure, and Achilles tendon lengthening is indicated if there is less than 5-degree dorsiflexion [20].

Another method of arthroereisis, also with beneficial results, describes drilling and implanting a screw into the lateral side of the talus rather than the calcaneus. Complications of this approach, according to a study by Scharer et al., include 15% reoperations due to implant migration, undercorrection, and overcorrection [19].

Tips and tricks

First, fluoroscopy should be used throughout the procedure to ensure the adequate position of the implant. Second, the lateral part of the implant should be at least 1 cm medial to the lateral edge of the calcaneus. Third, overstuffing of the sinus tarsi should be avoided. Finally, the guidewire should be clamped on the medial side to avoid backing out during trials.

Implant removal and position of the foot post-removal

The chief cause prompting the removal of an arthroereisis implant is postsurgical pain in the region of implantation. In adults, for instance, the incidence of sinus tarsi pain has been reported in as many as 46% of cases. Many times, the pain was severe enough to warrant implant removal. However, a notable and intriguing benefit to arthroereisis is the minimal, if any, loss of flatfoot correction after removal [21]. Chong et al. described two patients who had their implant removed due to persistent implant pain [22]. While implant pain was improved upon removal, the improved foot position was noted to be maintained in both patients. It had proposed that the screw not only acts as a mechanical wedge to support the talus into place but also stimulates the patient to correct their own flatfoot by modifying the proprioceptive pathways in the ankle [22]. Similarly, a case report by Needleman also found no significant difference in correction after eight months of follow-up post-removal of the arthroereisis implant [16]. If for a multitude of reasons an implant must be removed, arthroereisis poses the advantage of high reversibility. Symptoms of many complications, especially pain, resolve entirely for a majority of patients following implant removal [23].

Procedural outcomes

Several systematic review articles have investigated the outcomes of subtalar arthroereisis in the pediatric population. We reviewed three recent systematic reviews on the outcomes of subtalar arthroereisis by Tan et al. [24], Metcalfe et al. [23], and Smith et al. [3]. Common means for gauging the success of the procedure include clinical outcomes, radiographic metrics, and patient-reported satisfaction. In the systematic review by Smith et al., clinical outcomes were gauged based on standardized surveys such as the Manchester-Oxford Foot Questionnaire and the American Orthopedic Foot and Ankle Score, which quantify metrics such as pain, function, and alignment [3]. These outcome scores showed marked improvement (Table 1). Pain, for example, as assessed by the Visual Analogue Scale, was reduced from 5.5 preoperatively to 1.4 postoperatively [3].

**Table 1:** Pre and postoperative clinical outcomes. VAS = Visual Analogue Scale (of pain); VAS-FA = Visual Analogue Scale Foot and Ankle; MOXFQ = Manchester-Oxford Foot Questionnaire; AOFAS = American Orthopedic Foot and Ankle Score; OAFQC = Oxford Ankle Foot Questionnaire for Children; -P = physical; -S = school and play; - E = emotional; -F = footwear

Outcome	VAS	VAS-FA	MOXFQ	AOFAS	OAFQC-P	OAFQC-S	OAFQC-E	OAFQC-F
Studies	3	2	1	3	3	3	3	2
Number of feet	53	56	12	52	155	155	155	130
Preoperative mean	5.5	70.3	55.3	57.6	66.9	86.1	83.6	69.9
Postoperative mean	1.4	85.1	34.3	80.4	72.8	90.0	90.9	80.7
Difference	−4.1	14.9	−21.0	+22.6	+5.9	+3.9	+7.2	+10.9

Another common method to gauge the success of the procedure is to measure radiologic criteria. Table 2 lists the four common radiologic criteria used to gauge improvement post-procedure, namely, the lateral talo-first metatarsal angle (Meary’s angle), calcaneal pitch, lateral talocalcaneal angle, and the AP talocalcaneal angle (Kite’s angle). We chose these criteria as they were the four metrics that were shared among all three systematic reviews. In all systematic reviews, these postoperative radiographic parameters were within the limits of the normal population.

**Table 2:** Pre and postoperative mean of radiographic outcomes. *Mean value within the reference range.

		Tan et al. [24]	Metcalfe et al. [23]	Smith et al. [3]	Normal range
Calcaneal pitch	Preoperative mean	12.5°	14.3°	12.2°	15°–20°
	Postoperative mean	15.7°*	16.5°*	15.7°*	
Meary’s angle	Preoperative mean	16.67°	−16°	17.1°	0°–10°
	Postoperative mean	5.27°*	0°*	7.4°*	
Lateral talocalcaneal angle	Preoperative mean	36.06°	40.45°	42.4°	25°–45°
	Postoperative mean	27.19°*	33.65°*	36.4°*	
Kite’s angle	Preoperative mean	28.82°	28°	31.2°	15°–25°
	Postoperative mean	18.13°*	20°*	24.5°*	

Previous studies have also demonstrated a reasonably high level of patient satisfaction. Tan et al. found that 66.8% of the patients rated it excellent, 22.1% rated it good, 5.0% rated it fair, and 6.0% rated it poor [24]. Metcalfe et al. found that 79-100% of patients were satisfied, 11.1% were reasonably satisfied, 7.4% were moderately satisfied, and 0-21% were dissatisfied [23]. Smith et al. reported that out of 231 patients, 79.9% rated their satisfaction as excellent, 14.9% rated as good or fair, and 5.2% as poor [3]. Studies have generally reported favorable outcomes and patient satisfaction levels with a reasonable risk profile, which will be reviewed in the following section.

Complications

While subtalar arthroereisis has certain benefits for primary and secondary flatfoot-related foot pain and stabilizing the subtalar joint into its proper orientation, it is not without its drawbacks. In a systematic review of 76 studies, complication rates ranged from 4.8% to 18.6%. Most notably, between 7.1% and 19.3% of these surgeries had an unplanned implant removal [23]. Some of the more common complications across all device types include undercorrection, resulting from undersized implants, sinus tarsi pain, and extrusion of the implant. Some less common complications include overcorrection due to oversized implants, synovitis, infection, and peroneal spasm [21]. Many cases of synovitis are likely caused by a reaction to silicone microparticles from silastic implants [25]. While silicone is prone to fragmentation over time due to shear forces in the joint, the use of metal instead of silastic may decrease the rates of implant degradation and the ensuing inflammatory reaction [26]. Nerve injury is also an avoidable complication that can be averted with meticulous procedural execution. Another common complication, sinus tarsi pain, can typically be resolved with implant removal [12]. A rare but potentially dangerous complication regarding mobility and recovery potential is talar neck fracture [27]. Depending on the severity of each complication, remedies may include replacing a missized implant for a larger or smaller one, removing the implant entirely in the case of persistent pain, or appropriate anti-inflammatory or antibiotic treatments. As seen in outcome comparison, due to risks inherent to all surgical interventions, all options should be considered, beginning with the least invasive.

Advantages

Many of the advantages of arthroereisis are related to its relatively low level of invasiveness and easy reversibility compared to other procedures. For example, osteotomy carries with it risks inherent with bone grafts and changing the biomechanics of the foot. Osteotomies can result in calcaneocuboid subluxation, non-union or delayed union, or sural nerve injury [6]. Arthroereisis also avoids the chronic risks of joint fusion, which causes severe loss of subtalar joint mobility, impairing the foot’s ability to adapt to uneven surfaces and increasing the load on surrounding joints [6]. A key advantage of arthroereisis is the possibility of implant removal in the case of any postoperative complications, as well as the aforementioned maintenance of flatfoot correction after the screw is removed [22]. In addition, patients are able to bear weight immediately after the procedure, leading to its ability to be performed on an outpatient basis. These factors also contribute to the ability of a patient to have arthroereisis performed bilaterally without significantly altering morbidity. The safety, cost-effectiveness, and less invasive nature of this procedure suggest that it may likely be an appropriate option for some patients.

## Conclusions

Arthroereisis has shown statistically significant improvement in the primary correction of flexible pes planus in pediatric patients and is effective as an adjunctive procedure in accessory navicular syndrome, tarsal coalition, and posterior tibial tendon dysfunction. While complications such as sinus tarsi pain and undercorrection occur in some patients, in cases of severely painful flatfoot refractory to non-surgical intervention, this surgery may be indicated after thorough discussion with the patient regarding all possible outcomes. Further, due to the high degree of reversibility, with adverse symptoms often resolving after implant removal, arthroereisis is considered a relatively safe procedure for persistent flatfoot pain. The subtalar arthroereisis surgery, nonetheless, requires future long-term prospective studies examining the biomechanics of the procedure and implant, as well as forthcoming complications. Given its current conventional use and potential for improvement, subtalar arthroereisis can undergo immense progress regarding establishing evidence-based clinical guidelines and reaching a better consensus on how to optimize this surgery.
